# Repetitive Elements in Humans

**DOI:** 10.3390/ijms22042072

**Published:** 2021-02-19

**Authors:** Thomas Liehr

**Affiliations:** Institute of Human Genetics, Jena University Hospital, Friedrich Schiller University, Am Klinikum 1, D-07747 Jena, Germany; Thomas.Liehr@med.uni-jena.de

**Keywords:** variable numbers of tandem repeats (VNTRs), microsatellites, minisatellites, small-scale repetitive elements (SSREs), chromosomal heteromorphisms (CHs), higher-order repeat (HOR), retroviral DNA

## Abstract

Repetitive DNA in humans is still widely considered to be meaningless, and variations within this part of the genome are generally considered to be harmless to the carrier. In contrast, for euchromatic variation, one becomes more careful in classifying inter-individual differences as meaningless and rather tends to see them as possible influencers of the so-called ‘genetic background’, being able to at least potentially influence disease susceptibilities. Here, the known ‘bad boys’ among repetitive DNAs are reviewed. Variable numbers of tandem repeats (VNTRs = micro- and minisatellites), small-scale repetitive elements (SSREs) and even chromosomal heteromorphisms (CHs) may therefore have direct or indirect influences on human diseases and susceptibilities. Summarizing this specific aspect here for the first time should contribute to stimulating more research on human repetitive DNA. It should also become clear that these kinds of studies must be done at all available levels of resolution, i.e., from the base pair to chromosomal level and, importantly, the epigenetic level, as well.

## 1. Introduction

In humans, like in other higher species, the genome of one individual never looks 100% alike to another one [1], even among those of the same gender or between monozygotic twins [2]. When comparing individuals, it seems to be a rule than an exception that there are many genetic differences that do not have obvious, meaning simply traceable, effects on the phenotype. Such genetic differences can be found at all levels of resolution when studying a genome, from the base pair to the chromosomal (i.e., cytogenetic) level and any other level in between [1]. Certainly, a species, including humans, is defined by the numbers of genes and chromosomes. However, while the normal chromosome number in humans has been determined to be 46,XX or 46,XY [3], the number and definition of ‘a gene’ are unclear, even in humans [4]. As nicely summarized in [4], studies on the human genome size in the 1990s suggested that the human genome contains 50,000–100,000 protein-coding genes (PTGs); the first sequence of the genome in 2001 contained ~25,000–30,000 PTGs. In 2004, 22,287 protein-coding genes and 34,214 transcripts were reported in the Ensembl human gene catalog. Since 2008, RNA-seq has further identified a sheer endless series of non-coding transcribed sequences, which are grouped into long non-coding RNAs (lncRNAs), antisense RNA and miscellaneous RNA. In 2018, ~20,000 protein-coding genes, ~15,000 pseudogenes and ~17,000–25,500 non-coding RNAs were identified [4]. In Table 1, the corresponding numbers are given as of 2021 [5,6,7,8]. Furthermore, there are variations in the euchromatic coding sequences of healthy individuals (i.e., different alleles), and much more variability has been described in non-coding sequences [1,2,9]. From an evolutionary standpoint, these differences are reserved for adaptations of a population to new environmental conditions [1,10]. However, the majority of repetitive DNA sequences have not been sequenced and/or are not identifiable by currently applied methods. According to a recent paper from 2020, there are still 783 unclosed sequence gaps dispersed over 150 Mb (GRCh38 assembly) [11]. Many of them are due to the technical limitations of present sequencing approaches, especially due to the limitations of data processing pipelines [11,12].

In humans, polymorphic DNA changes have been reported at the base pair, kilobase pair, megabase pair and/or chromosomal levels in euchromatin (non-repetitive DNA) as well as in heterochromatin (repetitive DNA). The following genetic polymorphisms, listed according to their sizes, have been classified [13]:

1. Single nucleotide polymorphisms (SNPs) (1 base pair exchanges);
2. Microsatellites (1–10 base pair repeats);
3. Small-scale insertion/inversion/deletion/duplication polymorphisms (invs/ins/indels/invdups) (1–50 base pairs in size);
4. Minisatellites (10–100 base pairs in size);
5. Small-scale repetitive elements (SSREs) (0.1–0.8 kilobase pairs in size);
6. Submicroscopic copy number variants (CNVs) (in the megabase pair range);
7. Chromosomal heteromorphisms (CHs) (in the several megabase pair range);
8. Euchromatic variants (EVs) (in the several megabase pair range).

However, as these eight classes have been introduced artificially, i.e., method based, there are overlaps between them. Among these eight classes, mainly euchromatin is considered class 1 (SNPs), class 3 (invs/ins/indels/invdups), class 4 (CNVs) and class 8 (EVs); these classes are not further discussed here (see elsewhere for further details [13]), as the focus of this paper is on repetitive elements in humans. 

## 2. Repetitive Elements in Humans

Microsatellites (class 2) and minisatellites (class 4), also defined as variable numbers of tandem repeats (VNTRs), together with class 5 (small scale repetitive elements (SSREs)) and class 7 (chromosomal heteromorphisms (CHs)), are regions in human genomes classified as comprising mainly repetitive DNA [13]. Even though such repetitive DNA constitutes up to 75% of the human genome [1], changes in DNA sequences or in copy numbers of repetitive units are normally considered as lacking any influence on the human phenotype; in particular, they are not generally thought to be associated with diseases [14]. However, as is outlined below, there are examples of disease-causing repetitive DNA variants and/or phenotype changes due to alterations in repetitive DNA. Thus, the view of the role of repetitive DNA in humans is currently under discussion, especially in light of the fact that non-coding RNAs (ncRNAs) are derived from such repetitive DNAs [1,13]. As shown in Table 1 (rows: lncRNA and small ncRNA/pir ncRNA), the scale of such ncRNAs varies 10–100 times, according to the used database [5,8]. 

The majority of repetitive DNAs do not have (known) phenotypic consequences; however, some disease-causing exceptions are known for almost all variants of repetitive DNAs (see below). This is not that surprising in light of current thoughts on how repetitive DNA can affect genomes and may contribute to fundamental biological functions, such as cell proliferation in the context of embryogenesis [15], age-related diseases [16] and tumorigenesis (lncRNA) [17,18]. Roles for repetitive DNA have been speculated earlier and have been shown more recently in the context of gene expression regulation [15,16,17,18,19], the expression of lncRNA in X-chromosome inactivation [19] and the three-dimensional architecture of the nucleus [20].

### 2.1. Variable Number of Tandem Repeats (VNTRs)

Microsatellites and minisatellites (defined as VNTRs) are 1 to ~10 bp and 10 to 100 bp repeats, respectively. They are mainly found in larger clusters in (peri)centromeric and (sub)telomeric regions but also dispersed along all chromosomes [1,13]. The designation ‘satellite’ originates from experiments in the 1970s: the isopycnic centrifugation of DNA led to a major peak and a few side peaks; the latter were called satellite peaks. DNA extracted therefrom was designated as satellite DNA [1]. As summarized in [1], satellite DNAs have been divided into several subgroups, as shown in Table 2. These belong to the aforementioned classes 2, 4 and 5 and are also involved in class 7: the formation of CHs. 

Minisatellites are 1 to ~10 bp repeats, which are species-specifically distributed along all chromosomes, and are frequently applied in the molecular cytogenetic characterization of newly discovered or cytogenetically poorly studied species [21]. In human genetics and forensics, microsatellites are analyzed with other intentions: as each human being has an (almost) unique microsatellite pattern, polymerase chain reaction-based analyses can be informative to study uniparental disomy, to perform paternity testing or to identify a perpetrator [22]. These microsatellites are still best understood as being repetitive polymorphic DNA that does not influence the phenotype (see also Section 2.2.1). 

The best known microsatellite may be the 6 bp repeat of the telomeric sequence 5′-TTAGGG-3′. The preferred model system for humans, the lab mouse (*Mus musculus*), carries large terminal telomeric repeat blocks, which are practically not affected by aging [23]. In contrast, telomeric repeats in humans are notably degraded in somatic cells over their lifetime [24]. While it seems to be common sense that “telomeres protect chromosome ends from degradation and inappropriate DNA damage response activation through their association with specific factors” [25], their role in aging at least is under discussion [26], as no person has died yet from ‘too short telomeres’. As is typical for microsatellites, telomeric repeats are not only found at the chromosomal ends; there are also interstitial telomeric sequences (ITSs). At least some of these ITSs are interpreted as remnants from chromosome end-to-end-fusions during evolution [27]. Overall, the function of telomeric microsatellites in the cell and possibly the aging phenotype has been identified. Thus, this is the first indication that microsatellites may not only be a meaningless vestige of nature, but necessary for the biology of each living (human) cell. 

Minisatellites consist of 10–100 bp repeats and are predominantly located at pericentric and subtelomeric regions but can also be found throughout the genome at thousands of different locations. Minisatellites, as in the case of microsatellites, are characterized by high mutation rates and high diversity in populations. A subgroup of minisatellites has even been shown to be hypermutable when cells are subjected to genotoxic agents [28]. 

#### Disease-Associated VNTRs

Microsatellites and minisatellites are known to be harmless or harmful to their carrier depending on their integration site/localization. On a molecular level, i.e., the DNA level, 3 bp repeats (trinucleotides) can be observed as either harmless microsatellites or, if extended too much, harmful, disease-causing events. The latter is rare and appears in inherited human diseases like Huntington’s chorea and other so-called trinucleotide-repeat diseases. During meiosis, these trinucleotide repeats may be amplified or reduced in size. When they exceed a certain number (the phenomenon is called anticipation), this leads to a structurally altered gene product and, in consequence, to a disease [29]. Similarly, human diseases associated with minisatellites can occur if their copy numbers exceed a certain threshold [30,31]. Thus, the definition of VNTRs as a playground of evolution has to be at least carefully revised. Exceptions from being harmless have to be expected, and this also means that these repetitive elements have possibly not yet been sufficiently considered as potential underlying causes of human diseases.

### 2.2. Small-Scale Repetitive Elements (SSREs)

The gain, loss and insertion of DNA, which is constituted by 0.1 to 8 kb repeats, are called SSREs. Such SSREs can be slightly to highly repetitive, but the majority of them is (individually) invisible in light microscopy, as they do not reach 5 Mb in size. The latter is the lower level of resolution in banding cytogenetics [1]. Of special interest in the present context of an RNA virus–caused pandemic [32], major parts of SSREs are possibly of retroviral origin. During evolution, they may have become ‘normal’ components of eukaryotic genomes [33]. These retroviral-origin DNA repeats are predominantly grouped into ‘long interspersed nuclear elements’ (LINEs: 6–8 kb unit length) and ‘short interspersed nuclear elements’ (SINEs: 0.1–0.4 kb unit length). Furthermore, there are long terminal repeats that account for 8.3% of human genomes (0.2–3 kb unit length) [34].

- LINEs are formed by a group of mostly truncated retrotransposons and constitute >20% of the human genome. Three types of LINEs have been identified: LINE1 (~516,000 copies), LINE2 (~315,000 copies) and LINE3 (~37,000 copies). In fact, in humans, there are ~100 active LINEs per genome, which can still amplify and integrate at new genomic sites, as they comprise reverse transcriptase [26,27,28].
- Furthermore, SINEs derived from another subclass of retrotransposons provide ~13% of the human genome, with the feature that they can normally only become transcriptionally active if induced during infection of the human carrier by multiple DNA viruses or supported by LINE1 elements [35,36,37,38].

‘Polymorphic mitochondrial insertions’ (NumtS) are another polymorphic nuclear DNA of eukaryotes; they can be understood as a result of ongoing integration of mitochondrial DNA into the eukaryotic cell’s nucleus. More than 1000 NumtS are known in humans thus far [39]. As the mitochondrial circular genome is ~16.5kb in size, NumtS are normally shorter but can be arranged in repeats. Here, it is important to note that mitochondria are remnants of endosymbiotic organisms living in eukaryotic cells. To the best of the author’s knowledge, it is not clear whether the integration of NumtS may be, at least in part, dependent on LINE1 [40]. However, recently, we identified an exceptional case of an insertion of a cytogenetically visible NumtS block in chromosome 14 in a healthy carrier [41]. 

Furthermore, there is a subset of human satellite DNA (Table 2) that also belongs to the SSREs. They are DNA stretches of ~170 bp in length, can be found in low-copy repeats along the whole chromosome and are concentrated around centromeres. As detailed in [1], they form higher-order repeat (HOR) units with hundreds to tens of thousands of repeats close to the centromere. Accordingly, satellite DNAs (including classes 2 and 4) make up about 8–10% of the human genome; e.g., α-satellites are annotated at 44,058 loci covering 0.1% of the genome [42]. Interestingly, although these α- and γ-satellite sequences have been cloned, sequenced and known for decades, the majority of them are not included in genome browsers. Their sizes are variable between individuals; however, the ranges of the regions that they span have been previously reported to be between ~0.1 and 5 Mb [1]. At least some of these satellite DNAs are transcribed into RNA, but their role is yet unresolved. The fact that α-satellite DNA, for example, is expressed under cellular stress supports the idea that the alteration of heterochromatic to transcriptionally active regions could be correlated with genomic instability and oncogenesis; further supporting this notion is the fact that the tumor suppressor *PKNOX1* inhibits such satellite expression. Thus, histone methylation is important for satellite DNA expression, too. As the methylation status is also altered by heat shock treatment, it is not surprising that α-satellite sequences in chromosomes 12 and 15 were proven to be expressed after a heat shock in 2004 [1]. 

Overall, it is also unclear if and what influence the gain or loss of such satellite-DNA-based SSRE stretches (variations may be up to several 10 Mb in size) may have on an individual. Such changes are still generally considered to have no consequences; however, this seems to be unlikely [1,43] and is controversial [43,44].

#### 2.2.1. Disease-Associated SSREs 

ncRNAs can be important for the chromatin state (epigenetic marking and 3D structure) or for the transcriptional regulation of protein-coding genes, or they may be an insignificant background process [42]. Thus, the majority of all aforementioned SSREs are generally easy to consider as polymorph DNA. As previously discussed for VNTRs, it is a matter of location and circumstances (as for SINEs, the presence or absence of specific viruses) whether these conditions lead to clinical problems for its carrier [38]. Adverse effects of SSREs are the following:

- To date, ~100 examples are known of diseases that are caused by LINE insertions and/or amplification, such as epithelial cell cancer or neurological disorders [45,46,47]. Additionally, the hypomethylation of LINES has been linked to chromosomal instability and altered gene expression in cancer and normal tissue types [48,49]. Similarly, >50 human diseases are associated with SINE activation [38]. As shown for ALU repeats, which are classified as SINEs, in an evolutionary context, their significance cannot be underestimated [42].
- For small NUMTs, the first hints of an association with disease were recently reported as the disruption of genes through their insertion [46,50]. Furthermore, mitochondrial diseases can be inherited via NUMTs not only via the maternal but also the paternal line: this has been shown in seven families [51].
- The possible influence of satellite DNA copy numbers on human fertility have been previously reported [44,45], while the influence of RNA derived from HORs is still not really understood [46,52].

### 2.3. Chromosomal Heteromorphisms (CHs)

**Figure 1:**
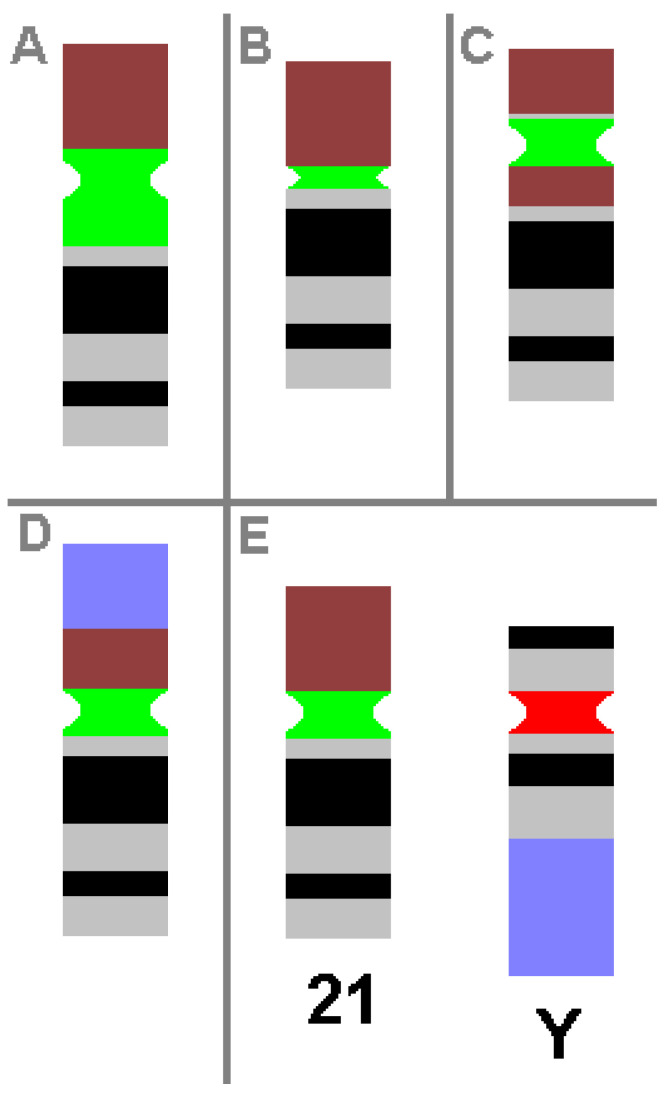
Schematic depiction of CHs, as known from cytogenetic diagnostics. Possible examples of centromeric amplification (**A**), centromeric diminution (**B**), pericentric inversion (**C**) and translocation (**D**) in chromosome 21 are shown, compared to a ‘normal’ chromosome 21 and a normal Y-chromosome (**E**). For clarity, the short arm of chromosome 21 is depicted in brown, the centromeric regions of chromosome 21 and Y-chromosome are in green and red, respectively, and the heterochromatic region of the Y-chromosome in the long arm is in blue. All aberrations shown do not cause any clinical problems.

CHs are karyotypic alterations frequently found within a certain percentage of the healthy population and are clearly visible under light microscopy. CHs include the gain, loss or inversion of cytogenetically visible heterochromatic material. CHs are constituted by micro- and minisatellites, α-, β- and other satellite DNAs, often organized in HORs [1]. In humans, such heteromorphic regions are located in the (peri)centric regions of all chromosomes, at the end of the long arm of the male Y-chromosome and in the short arms of acrocentric chromosomes (chromosomes 13, 14, 15, 21 and 22). The repeat units of HORs are similar, with 95–100% identity. Within these HORs, *α*-satellite monomers are often intermingled with other repeats, like SINEs, LINEs, LTRs or β-satellites [46]. The hundreds of different possible human CHs are summarized elsewhere and in Table 3 [1,53]. Additionally, some examples of CHs are shown in Figure 1.

Even though CHs are mainly considered a cytogenetic diagnostic problem, in exceptional cases, they can be useful in terms of the following:

- determination of paternity;
- differentiation of mono- and dizygotic twins;
- determination of the parental origin of derivative chromosomes or of haploid sets in polyploidy or chimera;
- detection of maternal cell contamination in amniotic fluid cell cultures;
- follow-up of bone marrow transplantations; or
- identification of some genetic linkages [1].

Finally, a special CH must be added here concerning the ‘polymorphism in chromosome numbers’. This is present in many species as a supernumerary ‘B’ chromosome (B), which is nicely defined by Ahmad and Martins as “extra karyotype units in addition to A chromosomes and found in some fungi and thousands of animals and plant species. Bs are uniquely characterized due to their non-Mendelian inheritance. A classical concept based on cytogenetics and genetics is that Bs are selfish and abundant with DNA repeats and transposons, and in most cases, they do not carry any function” [54]. In humans, the existence of Bs is under discussion. Some of the so-called small supernumerary marker chromosomes (sSMCs) could be candidates for human Bs [55,56]. About 50% of sSMCs only carry heterochromatic DNA, which is also amplified in CHs. 

#### Disease-Associated CHs

In the early era of cytogenetics, CHs were incorrectly associated with certain human diseases [1]. However, interestingly, recent work now suggests that CH amplification, like that of 1q12, could play a role in schizophrenia susceptibility [57]. Moreover, the presence of pure heterochromatic de novo sSMCs is thought to be a potential hint on uniparental disomy of the sSMC’s normal sister chromosomes [55,56]. Here, it is stressed that, like for the aforementioned polymorphisms, one needs to be careful in calling such alterations inert.

## 3. Conclusions

So-called genetic polymorphisms, present as variations in repetitive DNA, are still widely considered to be fairly meaningless and harmless to the carrier. However, as summarized here, there is more and more evidence that phenomena like VNTRs, SSREs and CHs (can) have an influence on human health, i.e., play a role in disease development and/or susceptibility. It is known that euchromatic variants, so-called allelic variants, like those in the ‘polymorphic’ ABO blood group system, can lead to phenotypic differences. In other words, it is better to be a carrier of blood group O than blood group A with respect to the genetic risk of developing thrombotic vascular and coronary artery disease during one’s lifetime [58,59]. While euchromatic variants are still the focus of mainstream research, VNTRs, SSREs and CHs and their influence on human disease and normal phenotypes have still not been sufficiently investigated [42], although single studies have occasionally, but not systematically, addressed this problem [11,12]. 

## Figures and Tables

**Table 1:** Number of genes in the human genome as of 2021.

Type	GENCODE [5]	NCBI [6]	Ensemble [7]	Genecards [8]
Total number of genes	60,660	54,644	59,662	270,168
Protein-coding genes	19,962	20,203	20,448	20,916
Genes that have more than one distinct translation	13,685 *	20,110 *	n.a.	n.a.
Non-coding genes	n.a.	17,871	23,997	219,587
lncRNA genes	17,958	n.a.	n.a.	75,839 *
Small ncRNA/pir ncRNA genes	7569	n.a.	n.a.	109,820 *
Pseudogenes	14,761	15,067	15,217	21,888
Others	350	1503	n.a.	7777

The fields with * indicate that they have not been summarized into the total number of genes.

**Table 2:** Satellite DNAs in humans.

Type	Length of Basic Units (bp)	Class of Genetic Polymorphisms
Satellite I DNA	17–25	4
Satellite II DNA	5	2
Satellite III DNA	5 interspersed with ~10 bp of definite sequence	2
α-satellite DNA	171	5
β-satellite DNA	68–69	4
γ-satellite DNA	220	5

**Table 3:** Number of different types of human heterochromatic chromosomal heteromorphisms (CHs) found in all chromosomes according to [53].

	Centromeric Amplification or Diminution	Pericentric Inversion	Others (e.g., Insertions or Translocations)
Number of types found	129	23	70

## Data Availability

Not applicable.

## Abbreviations

CHs	chromosomal heteromorphisms
CNVs	submicroscopic copy number variants
EVs	euchromatic variants
HOR	higher-order repeat
invs/ins/indels/invdups	small-scale insertion/inversion/deletion/duplication polymorphisms
ITSs	interstitial telomeric sequences
LINEs	long interspersed nuclear elements
lncRNAs	long non-coding RNAs
ncRNAs	non-coding RNAs
NumtS	Polymorphic mitochondrial insertions
pir	PIWI-interacting RNA
PTGs	protein-coding genes
SINEs	short interspersed nuclear elements
SNPs	single nucleotide polymorphisms
sSMC	small supernumerary marker chromosomes
SSREs	small-scale repetitive elements
VNTRs	variable number of tandem repeats
